# Supplementary material for: Hundreds of dual-stage antimalarial molecules discovered by a functional gametocyte screen
Source: Nat Commun. 2017 May 17;8:15160. doi: 10.1038/ncomms15160 (PMC5442318; doi:10.1038/ncomms15160)
Supplement: Supplementary Information — Supplementary Figure, Supplementary Table, Supplementary Note and Supplementary References [file ncomms15160-s1.pdf]

Supplementary Figure 1. TCAMS chemical series previously characterized at GSK that were progressed in the *Pf* FGAA screen. Scaffolds that were previously identified by GSK as promising antimalarial schizonticidal starting points are depicted in blue and compounds closely related to the GSK Published Kinase Inhibitor Set are depicted in green. One scaffold that belongs to both groups is shown in pink.

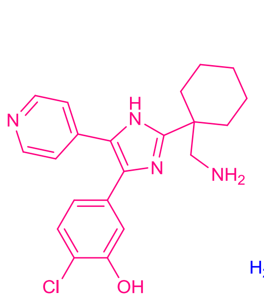

TCMDC-141154
CLUSTER 4 (12)

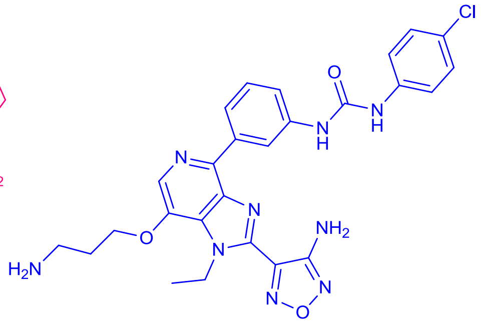

TCMDC-136152
CLUSTER 5 (8)

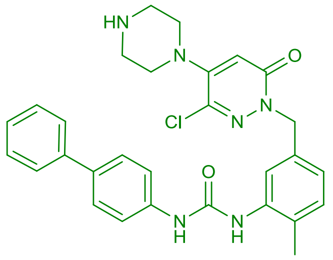

TCMDC-141698
CLUSTER 11 (6)

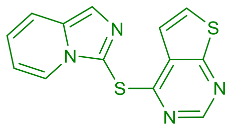

TCMDC-124602
CLUSTER 13 (6)

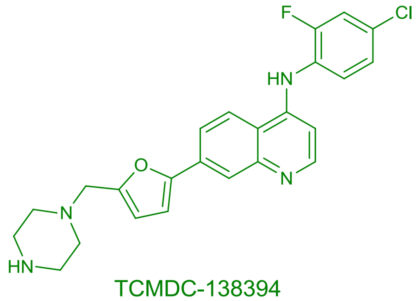

TCMDC-138394
CLUSTER 21 (5)

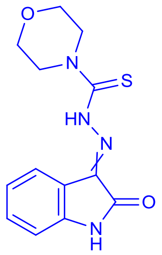

TCMDC-124263
CLUSTER 26 (9)

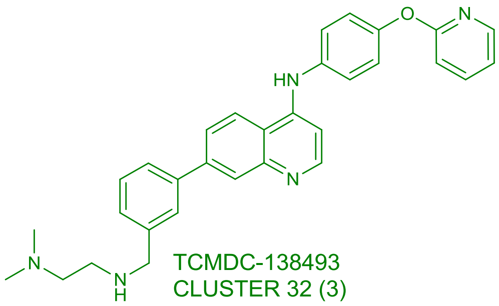

TCMDC-138493
CLUSTER 32 (3)

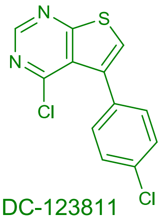

TCMDC-123811
CLUSTER 37 (3)

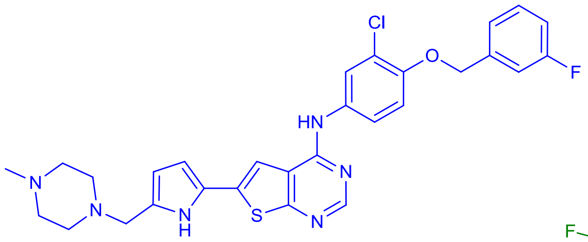

TCMDC-135458
CLUSTER 43 (3)

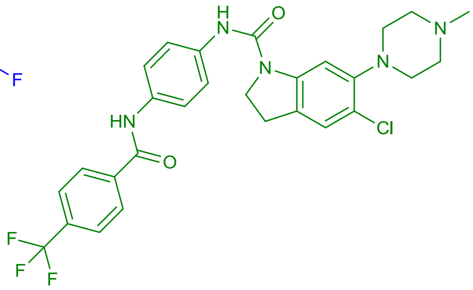

TCMDC-139270
SINGLETON 13

Supplementary Table 1. Results with raw data obtained from each independent experiment performed with the six compounds tested in the SMFA.

Paremeter Observed	TCMDC-141070			TCMDC-124559			TCMDC-125345		TCMDC-141698		TCMDC-123767		TCMDC-141154	
	Expt 1	Expt 2	Expt 3	Expt 1	Expt 2	Expt 3	Expt 1	Expt 2	Expt 1	Expt 2	Expt 1	Expt 2	Expt 1	Expt 2
Total Number of Fed Mosquitoes in Test Compound	44	45	38	38	47	53	21	41	43	46	38	62	53	60
Total Number of Fed Mosquitoes in DMSO Control	23	51	55	23	51	55	23	55	54	67	54	67	54	67
Prevalence of Infection in DMSO Control	78.2	70	91	78.2	70	91	78.2	91	78	88	78	88	78	88
Mean Oocyst Intensity in DMSO Control	4.6	1.8	4.6	4.6	1.8	4.6	4.6	4.6	5.1	5.3	5.1	5.3	5.1	5.3
Prevalence of Infection in Test Compound	0	15.4	0	74.29	70	79.17	35.97	38.22	14.04	8.8	13.26	13.2	5.46	6.16
Mean Oocyst Intensity in Test Compound	0	0.2	0	4.1	2.5	2.8	0.7	1.0	0.2	0.2	0.1	0.1	0.1	0.1
Mann Whitney Test, two tailed	p<0.0001	p<0.0001	p<0.0001	p=0.5477	p=0.0215[#]	p=0.0069	p=0.0012	p<0.0001	p<0.0001	p<0.0001	p<0.0001	p<0.0001	p<0.0001	p<0.0001
Statistical Significance	****	****	****	ns	*	**	**	****	****	****	****	****	****	****
Exflagellation Inhibition %	75	99	99	28	0	60	47	50	84	87	88	88	97	97
Oocyst Reduction Intensity %	100	92	100	24	0	39	88	83	97	97	97	97	99	98
Block in Transmission %	100	78	100	5	0	13	54	58	82	90	83	85	93	93

The Mann Whitney Test was used to compare the difference between the median oocyst instensity in the DMSO Control versus the Test group and treatments which show a statistically significant distribution are represented with asterisks: p<0.1, p<0.01, p<0.001, p<0.0001 is represented by *, **, ***, **** respectively. The exflagellation inhibition, block in transmission and reduction in oocyst intensity are calculated using the formulas provided in Supplementary Note 1. All data were analyzed using Microsoft Excel and GraphPad PRISM. # p value represents an increase in oocyst mean.

Supplementary Note 1. Formulas used to calculate raw data from SMFA experiments (Supplementary Table 1).

Exflagellation Inhibition was calculated using the following formula:

$$EI\% = 100 - \left(\frac{E_T * 100}{E_C}\right)$$

EI% = Exflagellation Inhibition Percentage
E_T and E_C are the number of exflagellating centers/field in test and control groups, respectively.

Transmission Blocking Potential was calculated using the following formulas:

$$OR\% = 100 - \left(\frac{OM_T * 100}{OM_C}\right)$$

OR%= Oocyst Reduction Percentage
OM_T = Oocyst Mean Intensity in Test group
OM_C = Oocyst Mean Intensity in Control group

$$BiT\% = 100 - \left(\frac{P_T * 100}{P_C}\right)$$

BiT%= Block in Transmission Percentage
P_T= Percentage of infected mosquitoes in the Test Group
P_C= Percentage of Infected Mosquitoes in Control Group

Supplementary References:

1. Gamo, F. J. *et al.* Thousands of chemical starting points for antimalarial lead identification. *Nature* **465**, 305-310, doi:10.1038/nature09107 (2010).
